# A Machine Learning Model for the Prediction of COVID-19 Severity Using RNA-Seq, Clinical, and Co-Morbidity Data

**DOI:** 10.3390/diagnostics14121284

**Published:** 2024-06-18

**Authors:** Sahil Sethi, Sushil Shakyawar, Athreya S. Reddy, Jai Chand Patel, Chittibabu Guda

**Affiliations:** 1 Department of Genetics, Cell Biology and Anatomy, University of Nebraska Medical Center, Omaha, NE 68105, USA; 2 Bond Life Sciences Center, University of Missouri, Columbia, MO 65211, USA

**Keywords:** COVID-19, severity prediction, machine learning, feature selection

## Abstract

The premise for this study emanated from the need to understand SARS-CoV-2 infections at the molecular level and to develop predictive tools for managing COVID-19 severity. With the varied clinical outcomes observed among infected individuals, creating a reliable machine learning (ML) model for predicting the severity of COVID-19 became paramount. Despite the availability of large-scale genomic and clinical data, previous studies have not effectively utilized multi-modality data for disease severity prediction using data-driven approaches. Our primary goal is to predict COVID-19 severity using a machine-learning model trained on a combination of patients’ gene expression, clinical features, and co-morbidity data. Employing various ML algorithms, including Logistic Regression (LR), XGBoost (XG), Naïve Bayes (NB), and Support Vector Machine (SVM), alongside feature selection methods, we sought to identify the best-performing model for disease severity prediction. The results highlighted XG as the superior classifier, with 95% accuracy and a 0.99 AUC (Area Under the Curve), for distinguishing severity groups. Additionally, the SHAP analysis revealed vital features contributing to prediction, including several genes such as COX14, LAMB2, DOLK, SDCBP2, RHBDL1, and IER3-AS1. Notably, two clinical features, the absolute neutrophil count and Viremia Categories, emerged as top contributors. Integrating multiple data modalities has significantly improved the accuracy of disease severity prediction compared to using any single modality. The identified features could serve as biomarkers for COVID-19 prognosis and patient care, allowing clinicians to optimize treatment strategies and refine clinical decision-making processes for enhanced patient outcomes.

## 1. Introduction

The global impact of the COVID-19 pandemic has warranted a robust and nuanced understanding of the factors influencing disease severity to improve clinical decision support and patient outcomes. With the emergence of advanced technologies, particularly in artificial intelligence (AI) and ML, a growing opportunity exists to harness the available data for predictive modeling and disease management. Previous studies have demonstrated the efficacy of these technologies in diagnosing and managing viral diseases, including COVID-19 [1,2].

The unique nature of COVID-19 infection and disease progression poses challenges for treatment development. While SARS-CoV-2 RNA tests diagnose infections qualitatively, the early determination of disease severity is crucial for devising an appropriate treatment strategy. Although CT scans and conventional laboratory procedures are helpful, they may not capture lung alterations in 20% of COVID-19 cases [3]. On the other hand, lab tests like blood cell counts offer practical alternatives, revealing reduced white blood cell and platelet counts alongside elevated serum ferritin and C-reactive protein levels in COVID-19 patients [4]. Clinical characteristics like the C-reactive protein amount, gender, age, lactic dehydrogenase, and lymphocyte count correlate significantly with COVID-19 severity [5]. RNA-based assessments, applicable across healthcare, are crucial in COVID-19 diagnosis and prognosis [6]. Gene expression patterns across patient populations, identified through RNA-seq data, can be explored to identify potential biomarkers for COVID-19 progression and severity [6,7]. On this front, ML emerges as a promising tool for precise and rapid disease severity assessment. ML algorithms, designed to uncover hidden patterns and intricate correlations, have been employed in various studies predicting contributing factors for COVID-19 severity [8,9,10].

Despite the efforts to leverage clinical and gene expression data for predicting COVID-19 severity, the current challenge lies in integrating genomic and clinical data to develop accurate prognostic models for effective disease management.

In this study, we devolved machine-learning models to predict COVID-19 severity by incorporating three data modalities: RNA-seq-based gene expression, diverse clinical features, and co-morbidity information. Combining these three data types aims to capture the correlations among the three modalities, enhancing disease severity prediction accuracy and offering accurate clinical decision support. Further, our study employs SHAP analysis and pathway enrichment techniques to unravel the contributing factors for prediction and the biological pathways involved in disease severity.

## 2. Materials and Methods

### 2.1. Datasets and Preprocessing

We obtained a GSE212041 dataset from the GEO database [11]. The dataset comprised 392 patients: 306 hospitalized COVID-19 patients, 78 symptomatic controls, and 8 healthy controls. From these patients, a total of 722 blood samples were collected at different time points: 374 samples on day 0 (D0), 212 samples on day 3 (D3), and 136 on day 7 (D7) from the COVID-19-positive patients admitted to the Massachusetts General Hospital Emergency Department (ED).

In the present study, we used data from only 299 COVID-19 patients out of 306 because the missing metadata for the remaining seven patients provided samples at D0. The original research classified patients into five classes (A1–A5) based on the severity of the disease (Table 1). Classes A1 and A2 included patients recognized as dead within 28 days and those who survived but required mechanical ventilation and intubation, respectively. We regrouped patients from these classes into a single group termed ‘severe’. Patients in the A3 class were placed in the ‘moderate’ group, while patients originally in A4 and A5 were placed in the ‘mild’ group (Table 1).

### 2.2. Data Description and Preprocessing

Gene expression data

All patients’ raw read count data underwent initial filtration, removing genes with expression values as zeros or NaN in over 20% of the samples. The total number of gene features after preprocessing was 5293 (Supplementary Table S1). Subsequently, the DEseq2 package was applied to normalize raw read counts, and FPKM values were computed using the FPKM function [12,13]. We also used an independent dataset (GSE172114) comprised exclusively of blood gene expression profiles (FPKM values) of 69 COVID-19 patients (46 critical and 23 non-critical) to test the performance of models.

Clinical data

The clinical data encompassed all 11 features, including age, body mass index (BMI), lactate dehydrogenase (ldh), absolute neutrophil count (abs_neut), absolute lymphocyte count (abs_lymph), cardiac event (Trop), Viremia, creatinine, absolute monocyte (abs_mono), D-dimer (ddimer), c-reactive protein (crp), and neutrophil enrichment (Neu). More details of the clinical features are provided in Supplementary Table S2.

Co-morbidity data

In addition to clinical features, co-morbidity data included nine variables describing pre-existing conditions such as heart disease (HEART), lung disease (LUNG), kidney disease (KIDNEY), diabetes (DIABETES), hypertension (HTN), immunocompromised conditions (IMMUNO), respiratory symptoms (Resp_Symp), febrile symptoms (Fever_Sympt), and GI-related symptoms (GI_Symp). More information about co-morbidity features is mentioned in Supplementary Table S3.

### 2.3. Data Augmentation

Data augmentation artificially increases the size or diversity of a dataset used for biological analysis. This technique is commonly employed in biological research, particularly in genomics, bioinformatics, and image analysis, where the control sample size is very low compared to the treatment sample size [14,15]. In the present study, we needed to balance the sample size for the ‘mild’ and ‘severe’ classes to be on par with that of the ‘moderate’ class (Table 1). We used Adaptive Synthetic Sampling (ADASYN) to oversample the minority class and address the class imbalance problem [16]. ADASYN mitigates this issue by adaptively generating synthetic samples for the minority class based on the local density distribution of existing instances [17]. The algorithm works mainly in four steps: (1) the data distribution analysis of all the classes, (2) the density estimation and identification of k-nearest neighbors of all instances in the minority classes, (3) the difficulty level measurement of minority and majority class instances, and (4) adaptive sampling based on the difficulty ratio to determine the number of synthetic samples needed for each minority class instance. In our experiments, we used default values of all parameters and hyperparameters such as, sampling_strategy: ‘auto’, n_neighbors: 3, details: n_jobs: 1, and random_state: None.

### 2.4. The Determination of Feature Weights and Integration

**Figure 1:**
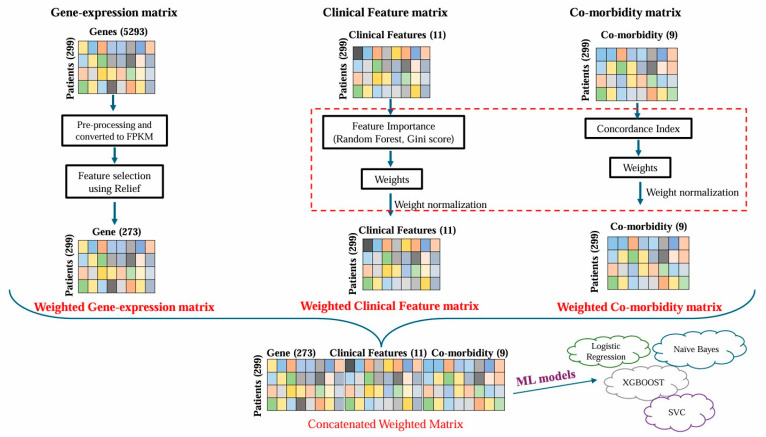
The workflow of preprocessing various data types, generating individual feature weight matrices and integrations, and machine learning model training for COVID-19 severity prediction.

In disease severity prediction, implementing feature weights plays a crucial role in enhancing the accuracy and interpretability of ML models. It assigns different levels of importance to various features within each data type, allowing the model to focus on the most influential factors in predicting disease severity. Below, we describe strategies for assigning and utilizing feature weights for each data modality before model training and severity prediction, as depicted in Figure 1.

#### 2.4.1. Weights to Gene Features

A LASSO (Least Absolute Shrinkage and Selection Operator) regularization approach was implemented for gene expression data to ascertain the correlation coefficients for each gene with the severity of COVID-19 [18]. All parameters were set as defaults with an alpha value of 1.0. This technique aids in identifying and emphasizing the genes that exhibit a significant impact on predicting disease severity. The model can prioritize their influence by assigning weights to these genes based on these expression values, contributing to a more refined and accurate prediction (Supplementary Table S4).

#### 2.4.2. Weights to Clinical Features

In this case, we calculated the Gini index, representing the importance of each clinical feature. This index, integrated with the Random Forest Classifier module, assigned weights to clinical features based on their predictive power [19]. Features deemed to be more critical in determining disease severity were assigned higher weights, ensuring that the model precedes these influential factors during prediction. Finally, a weighted clinical feature matrix was generated, as illustrated in Figure 1. The clinical features and their corresponding weights are provided in (Supplementary Table S5).

#### 2.4.3. Weights to Co-Morbidity Features

The impact of pre-existing conditions on COVID-19 severity was assessed using the Python library Lifelines, which calculated the concordance index (CI) [20]. The CI, representing the weight of each pre-existing condition, was then integrated into the original matrix to create a general final weighted co-morbidity matrix (Figure 1, Supplementary Table S6). By assigning weights to different medical conditions, the model could discern their relative contributions to the overall prediction of COVID-19 severity.

#### 2.4.4. Integration of Weighted Feature Matrices

The weighted gene expression, clinical, and co-morbidity data were concatenated to generate a final integrated matrix, which was used as the input for the ML model, as shown in Figure 1. Including feature weights ensured that the model considered the varying importance of genes, clinical indicators, and pre-existing conditions when predicting disease severity. This approach allowed for more refined and accurate prediction, as the model assigned higher importance to features with greater predictive power.

### 2.5. Machine Learning Model

Four distinct ML algorithms, including LR, XG, NB, and SVMs, were employed to identify a robust prediction model for disease severity [21,22,23,24]. These are the most used algorithms for classification problems due to their strengths and adaptability to different data types. LR is well-suited for binary or multiclass classification with interpretable results, while XG excels in boosting decision trees for improved predictive performance. NB is effective in probabilistic classification, particularly with relatively simple and independent features. On the other hand, an SVM is powerful for finding optimal hyperplanes in high-dimensional spaces and is useful in scenarios where complex decision boundaries are needed. ANN, conversely, can capture intricate patterns and non-linear relationships in data, making them suitable for tasks demanding high complexity and abstraction. Exploring these diverse algorithms allows for a comprehensive exploration of the data’s characteristics and the potential to achieve better overall model performance. Ten-fold cross-validation was used for all models.

The Scikit-learn libraries were employed to import these classifiers (Scikit-learn Machine Learning in Python) [25]. At first, we applied LR, recognized as a heuristic method for multi-class classification. The LR algorithm was implemented using the Scikit-learn library’s Logistic Regression module, utilizing default parameters while specifying the ‘OvR’ mode (One-vs-Rest) for the multiclass parameter. The algorithm XG was executed through the XG Python library. The algorithm was configured with a learning rate of 0.5, a maximum tree depth of 3, and 800 runs (n-estimators) for learning. The NB was implemented with its default parameters of class_count as three and class_prior as ‘none’. The SVM classifier algorithm was also applied with all default settings (C = 1.0, kernel = ‘rbf’, degree = 3). Finally, an ANN was implemented with three layers, 100 epochs, ReLU (Rectified Linear Unit), and SoftMax as activation layers, Adam as the optimizer, and Categorical Cross-Entropy set as the loss function.

### 2.6. Evaluation of Model Performance and Comparison

We evaluated the model’s performance by measuring the accuracy, F1 score, and the AUC. We used the cross_value_score function from Scikit-learn Python to calculate the evaluation metrics.

### 2.7. Feature Importance and Contribution Analyses

We adopted SHapley Additive exPlanations (SHAPs), commonly used to explain the output of any ML model in the context of the feature’s contributions. Because of the different combinations of input features, Shapley was utilized to find features with high classification power between COVID-19 severity groups [26]. In the context of gene expression data, SHAP helps discern the impact of individual genes on predicting disease severity. For clinical features, the impact of variables such as age, neutrophil count, and other clinical indicators on prediction can be identified. Similarly, it elucidates the influence of pre-existing conditions on the overall severity prediction. We used a combined (gene-expression, co-morbidity, and clinical feature matrix) input matrix in SHAP with 299 rows (patients) and 294 columns (features). By integrating SHAP values across these three different data types, a comprehensive understanding of feature contributions is attained, facilitating the interpretation of ML model predictions and enhancing the model’s transparency and interpretability.

### 2.8. Downstream Analysis of Significant Gene Features

We performed pathway enrichment analysis using 2753 significant gene features obtained after applying feature selection using LASSO regression. All the significant genes were used as input for Ingenuity Pathway Analysis (IPA) with default parameters [27]. Enriched biological pathways were observed to understand their associations with the severity of COVID-19.

## 3. Results

This study seeks to employ ML models to predict disease severity and identify the associated clinicogenomic features in COVID-19 patients. We analyzed the gene expression data and the clinical and co-morbidity information of 299 hospitalized COVID-19 patients. After preprocessing the data, we had 253 gene features, 11 clinical features, and 9 co-morbidity features for all the patients, as mentioned in Supplementary Tables S3 and S4. In the gene expression dataset, our feature selection strategy identified 2753 genes that were most relevant and highly associated with disease severity. These genes and the clinical and co-morbidity features were further used as input in model training. Multiple machine learning algorithms, including LR, NB, XG, and SVM models, were trained to classify the severity classes of ‘severe’, ‘moderate’, and ‘mild’. We used F1 and accuracy metrics to evaluate each model’s performance. The schematic workflow of the data integration approach, feature selection, and model development is provided in Figure 1.

### 3.1. Effects of Data Augmentation on Model Performance

As the method mentions, ADASYN oversamples the ‘severe’ and ‘mild’ groups to address the class imbalance. This experiment used only gene expression data due to its rich feature size. As a result, the number of samples was increased from 76 to 120 in the ‘severe’ class and 74 to 134 in the ‘mild’ class after augmentation (Table 2). ADASYN automatically determines the augmentation size of the minority classes to bring them up to par with the majority class.

We evaluated LR, XG, NB, and SVM performances before and after augmentation. As shown in Table 3, the augmented model demonstrates a noticeable improvement in accuracy and the AUC compared to the original models. XG achieved a remarkable enhancement from a 40% accuracy and an AUC of 0.47 to a 95% accuracy and a 0.99 AUC after data augmentation. In comparison, LR demonstrated a slight increase in accuracy from 43% to 81% and an AUC from 0.56 to 0.93. Similarly, NB and the SVM showed slight improvement after data augmentation (Table 3). In this, we observed that increasing the size and diversity in the training dataset allowed the model to encounter more features and generalize better to test data. More specifically, the strategy introduced noise and variation in the classes of “Severe” and “Mild”, which, in a true sense, helped prevent the model from fitting to the noise in the training data and improved its ability to generalize to new and unseen examples.

### 3.2. The Evaluation of ML Models with Single- and Multi-Modality Data

In earlier stages, data augmentation only contributed to marginal improvements in class predictions for a limited number of models. This raised concerns about the potential misallocation of feature weights during model training, leading to suboptimal performance even after oversampling. Therefore, we calculated weights for each feature and generated individually weighted matrices for each data type (i.e., gene expression, clinical, and co-morbidity) and subsequently used them as input for the model. As mentioned in the methodology, the Gini index score, the concordance index, and the R-squared score from LASSO regression were used to calculate weights to corresponding features in each data matrix, i.e., the clinical, co-morbidity, and gene expression data matrices. The assignment of weights to feature matrices is a critical aspect influencing the performance of predictive models. By assigning different weights to individual feature matrices, the model learns to prioritize and emphasize specific types of information. The complete set of utilized clinical and co-morbidity data can be found in Supplementary Table S7.

**Figure 2:**
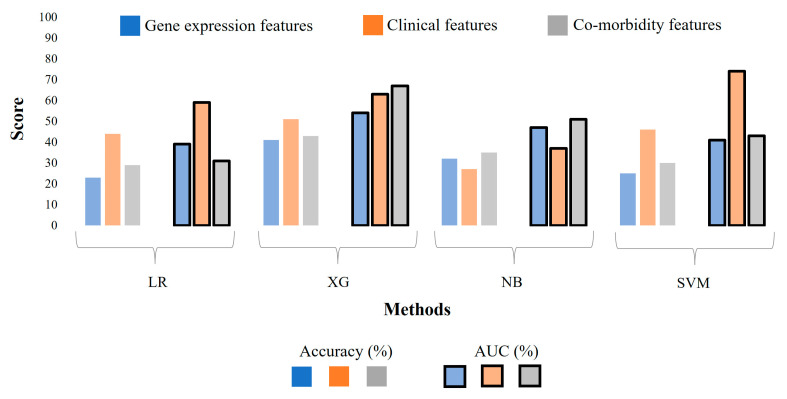
The evaluation of ML models with 10-fold cross-validation when individual data types are used as input. LR: Logistic Regression, XG: XGBoost, NB: Naïve Bayes, SVM: a Support Vector Machine.

**Figure 3:**
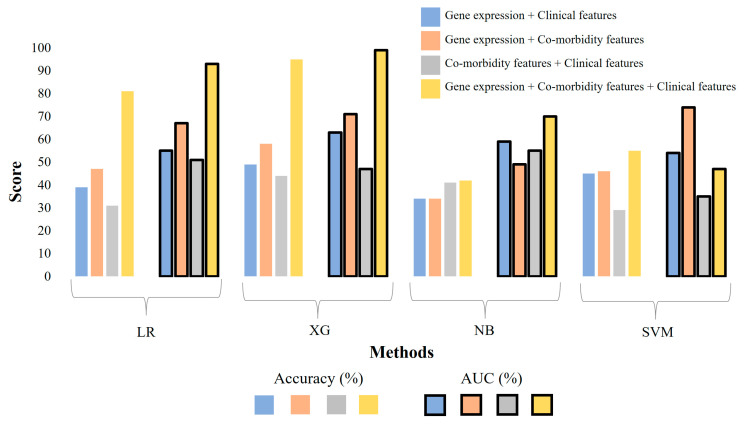
The evaluation of ML models with 10-fold cross-validation when different combinations of data types are used as input. LR: Logistic Regression, XG: XGBoost, NB: Naïve Bayes, SVM: a Support Vector Machine.

As shown in Figure 2, the 10-fold accuracies for ML models generated from single-modality-weighted matrices are low for all algorithms, indicating that the features were insufficient for the ML Model to predict the difference between the three COVID-19 groups. Additionally, we evaluated our model using an independent dataset (GSE172114), consisting solely of blood gene expression profiles from 69 COVID-19 patients (46 critical and 23 non-critical). The preprocessing procedure mirrored that of GSE212041. In this experiment, XG demonstrated superior performance, achieving a peak accuracy/AUC of 75%/0.87. In comparison, the original XG model trained on dataset GSE212041 (gene expression only) achieved lower accuracy and AUC of 41 and 0.54 (Figure 2), respectively. Other classifiers, such as Naive Bayes, exhibited the lowest accuracy and AUC of 46% and 0.51, respectively, to identify “critical” and “non-critical” cases. The LR and SVM models yielded accuracy/AUC values of 50%/0.64 and 57%/0.71, respectively. We further utilized different combinations of the multi-modality weighted matrices as input for ML models, which showed increased prediction accuracies across the board (Figure 3). Combining two data modalities has significantly improved the accuracy of all ML models except for the SVM, and combining all three data modalities has substantially increased the accuracy in all cases except for the SVM. Specifically, the XG algorithm attained an accuracy of 95% and an AUC of 0.99, making it the top-performing algorithm for distinguishing between the three severity groups (‘severe’, ‘moderate’, and ‘mild’) of COVID-19 patients (Figure 3).

### 3.3. The Evaluation of Model Performance Using Different Weight Combinations for Data Modalities

**Figure 4:**
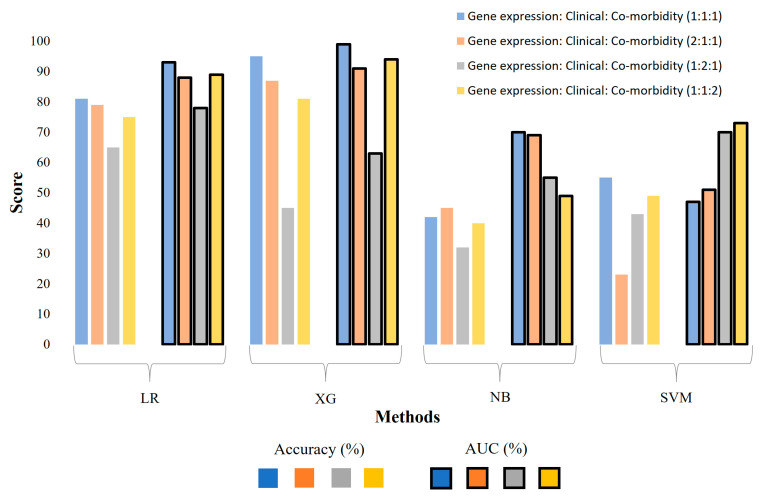
The evaluation of machine learning models using different combinations of weights for the three data modalities. The numbers in parenthesis represent the proportions of weights used for each modality in the data matrices used for model building. LR: Logistic Regression, XG: XGBoost, NB: Naïve Bayes, SVM: a Support Vector Machine.

To investigate the optimal combination of weights for each data modality, we assigned different weights to each data matrix, followed by concatenation to generate an integrated matrix used as input for the model. Gene expression, clinical features, and co-morbidity matrices were weighted at 1:1:1, 2:1:1, 1:2:1, and 1:1:2 proportions to build the corresponding models. Interestingly, the model with an equal weightage (1:1:1) for all data modalities produced the highest accuracy of 95% and an AUC of 0.99 using XG (Figure 4). A similar trend was observed with LR and NB models with corresponding weight combinations; however, the SVM models showed a different trend, with the highest AUC observed in the 1:1:2 model. The comparison of predictive performance among these models reveals the impact of different combinations of feature matrices on the overall model effectiveness. Models with various combinations of weights for each data modality unveil the relative importance of molecular, clinical, and co-morbidity data in the overall performance of the models and help optimize the ML models for the best performance.

### 3.4. Feature Importance Analyses

**Figure 5:**
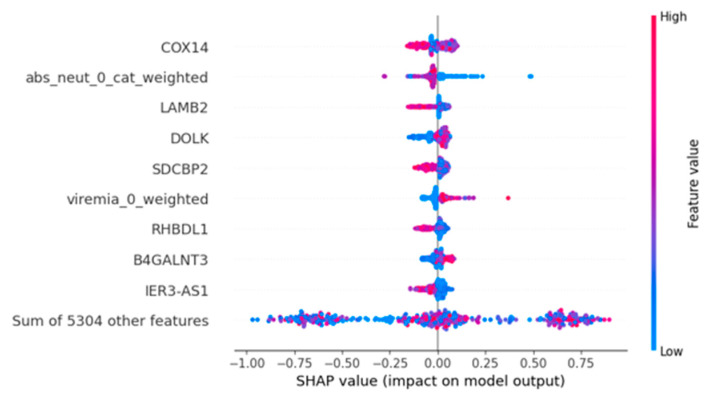
A beeswarm plot, ranked by mean absolute SHAP value. This provides a rich overview of how the variables impact the model’s predictions across all data. The input variables are ranked from top to bottom by their mean absolute SHAP values.

After determining XG to be the best-performing model and optimizing the weight combination for different data modalities (1:1:1), we sought to identify the contributions of individual features to predicting disease severity. We used the SHAP method, which provided the SHAP score for each feature used in the model training [26]. This score ranges from −1 to +1 and represents the significance of each feature and its effect on the model’s performance for predicting COVID-19 severity. The beeswarm plot shows how each SHAP feature positively or negatively contributes to the model prediction (Figure 5). The points are distributed horizontally along the x-axis according to their SHAP value, reflecting the strength of a feature’s impact on the model’s output. The color of the dot represents the original value of the feature, in an instance, with red representing a high value and blue representing a low value. The points are stacked vertically in places with a high density of SHAP values. Examining the color distribution horizontally along the x-axis for each variable provides insights into the general relationship between a variable’s original value and its SHAP value. The topmost gene expression features significantly affecting the model’s accuracy are COX14, LAMB2, DOLK, SDCBP2, RHBDL1, and IER3-AS1 genes from the RNA-seq data. The absolute neutrophil count and Viremia were identified among the clinical features, but no co-morbidity features stood out in the SHAP analysis (Figure 5). We see a dense cluster with low correlation with small-but-positive SHAP values for DOLK. LAMB2 extends further towards the left, suggesting LAMB2 has a stronger negative impact on COVID-19. The top gene features from SHAP can be further analyzed to understand the enriched pathways associated with the top contributing genes.

### 3.5. The Pathway Enrichment Analysis of Top Contributing Genes

**Figure 6:**
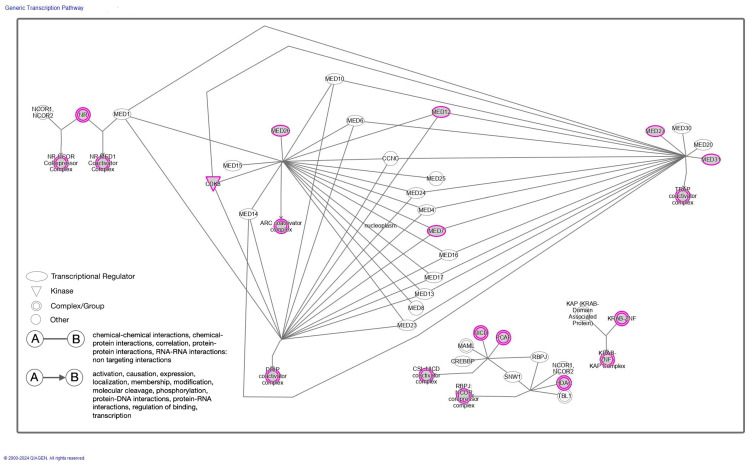
The canonical generic transcription pathway was enriched in the top 25% highest-scoring features based on SHAP scores.

Based on SHAP scores, we selected the top 25% (1324) of contributing genes (Supplementary Table S8) and subjected them to pathway enrichment analysis using IPA. This analysis revealed several significantly enriched pathways, shedding light on the severity of key molecular processes associated with COVID-19. The top five canonical pathways are shown in Table 4. The generic transcription pathway is the topmost pathway. Several biochemical pathways, such as the generic transcription pathway, are key to understanding the host–pathogen interactions during a SARS-CoV-2 infection in the nucleoplasm, impacting etiology, pathogenesis, or prognosis (Figure 6). The assembly involving nuclear receptor (NR) protein(s), CDK8, and MED proteins, forming the TRAP coactivator complex [TRAP coactivator], may modulate transcription factors and other proteins that are vital in the host’s immune response, potentially affecting the prognosis of COVID-19 [28] (Table 4). The second pathway is ‘immunoregulatory interactions between a lymphoid and a non-lymphoid cell’ that may involve interactions between SARS-CoV-2 and immune cells during COVID-19 pathogenesis. This pathway triggers HLA interactions with the KLRC1 complex and KLRF interactions with the CLEC2B dimer [29]. The virus then infects various immune cells, including lymphoid cells such as T lymphocytes, leading to the dysregulation of immune responses [30] (Supplementary Figure S1). The next one is the ‘mitotic prometaphase pathway’, where the dysregulation of mitosis can lead to cellular stress and affect tissue homeostasis. In this pathway, phosphorylated p-T2055-NUMA1 homodimer binds to nucleated microtubules in the cytoplasm. Mitotic kinase, CCNB1 phosphorylates Condensin I complex, forming phosphorylated CDK1 Phosphorylated Condensin I. PLK1 catalyzes the phosphorylation of STAG2, the RAD21-Ac-Cohesin: PDS5:CDCA5: WAPAL complex at centromeres, affecting sister centromeres and microtubule interactions, which in turn contributes to the pathophysiology of COVID-19 in various organs [31] (Supplementary Figure S2). The fourth pathway is FCGR-dependent phagocytosis, reflecting the role of Fcgamma receptors (FCGR) in mediating phagocytosis by binding to antibodies and opsonizing viral particles. The phosphorylated clustered PLCG complex in the plasma membrane yields the PI (3,4,5) P3 and p-PLCG complex. Moreover, the branching complex in the cytoplasm forms the ARP2/3: actin: ADP complex and activates WAVE2, WASP, and N-WASP proteins [32] (Supplementary Figure S3). The last one is the ‘cilium assembly pathway’ that COVID-19 may impact in respiratory epithelial cells. Multiple proteins in cilia form the IFT-B complex for intraflagellar transport, and the BBS/CCT complex catalyzes the assembly of the BBSome complex in the cytoplasm for ciliary function, affecting the clearance of mucus and pathogens from the airways [33] (Supplementary Figure S4). Overall, COVID-19’s impact on these pathways and processes reflects its complex interactions with host cells and the immune system, contributing to the diverse clinical manifestations and outcomes observed in infected individuals. Understanding these connections is critical for developing targeted therapies and interventions against the virus.

## 4. Discussion

ML models have been widely used on COVID-19 data to improve risk predictions for hospitalization and critical disease outbreaks [34,35,36]. Despite the numerous ML models that have been built, there are very few studies in which the models tried to use both clinical and genomic data to predict the severity of COVID-19 [37,38]. Hence, the project aims to develop a prognostic ML model to predict the severity of COVID-19 based on gene expression and clinical and co-morbidity data. We used data augmentation to balance the class sample size, explored various ML models to identify the best-performing model, and optimized the ML model’s performance using different weights. In addition, we used the SHAP score to find the features that contribute the most to the model’s performance (Figure 5).

Four machine learning algorithms, LR, XG, NB, and SVMs, were used to initially build a classification model only based on the normalized gene expression data from COVID-19 patients that belong to three severity groups, ‘mild, moderate, and severe’ (Table 1). To avoid overfitting the ‘moderate’ group with the same sample size as the other two groups combined, we augmented and balanced the sample size of the minority classes using ADASYN (Table 2). Models built from balanced datasets have shown significantly improved performance (accuracy and AUC) for all ML methods compared to those using unbalanced datasets (Table 3). Only gene expression features were used for the initial testing of ML models as this data modality has thousands of data points compared to merely twelve and nine features in the clinical and co-morbidity modalities, respectively.

We have built separate models for each data modality, their pair-wise combinations, and all three combined. The integration of the three data modalities showed a significant improvement in the predictive power of the ML models compared to those using a single modality or pair-wise data modalities (Figure 2 and Figure 3), with the accuracy reaching 95% and an AUC of 99% for the XG model that was trained with all three modalities. Our results align with the other studies highlighting the importance of using integrated multi-omics data in predictive models to leverage the synergistic effect of combining different data modalities. For example, ML models integrating transcriptomic and clinical data for predicting the clinical outcomes of COVID-19 patients showed enhanced accuracy [39]. In addition, the XG algorithm outperformed the other classifiers because it implemented a gradient-boosting framework, allowing it to build decision trees sequentially and optimize for bias and variance. Incorporating regularization techniques, such as L1 and L2 regularization, effectively prevents overfitting [40].

Furthermore, the most important features with the highest predictive power in the integrated model were shapely identified. The COX14 gene was identified as the top feature, significantly contributing to the model’s predictive power. The COX14 gene (cytochrome c oxidase; COX) encodes a core protein of the mitochondrial electron transport chain’s complex IV assembly, a vital component of the COX protein’s catalytic core, essential in electron transport [41]. A recent proteomic study of COVID-19 patients suggested elevated levels of the components of cytochrome c electron transport complexes in the plasma of COVID-19 patients compared to that of the normal controls [42]. The second most important feature from the SHAP analysis, an absolute number of neutrophil counts, emerged from the clinical feature set. Several studies reported high levels of neutrophils in severe COVID-19 patients and neutrophil-related cytokines like IL-8 and IL-6 [43,44,45]. Neutrophils detect single-stranded RNA viruses like SARS-CoV-2 because they express multiple Toll-like receptors: TLR7, TLR8, and TLR9. Once the TLR receptors are activated, other physiological processes, such as NF-κB and interferon regulatory factors, are activated (IRF7) [46]. The latter activation process produces chemokines and pro-inflammatory cytokines in neutrophils that induce pulmonary infiltration and hyperinflammation in COVID-19 patients [47].

Furthermore, the LAMB2 gene was also identified among the top three features in our SHAP analysis. This gene encodes the basement membrane protein laminin β2, part of the heterotrimeric laminin isoforms [48]. LAMB2 was identified as a diagnostic biomarker for COVID-19 based on a bioinformatics analysis of the gene expression dataset of COVID-19 patients [49]. Moreover, our findings underscore the significance of specific pathways enriched in the top 25% of genes identified through SHAP values. Pathways include generic transcription, immunoregulatory interactions between a lymphoid and non-lymphoid cell, mitotic prometaphase, FCGR-dependent phagocytosis, and cilium assembly. In a SARS-CoV-2 infection, fundamental host cellular processes such as generic transcription and immune responses are expected to be perturbed. Some of the genes involved in these processes could indicate disease progression and severity.

The super pathway of Inositol Phosphate Compounds involves genes responsible for inositol production, which is essential to generate the phosphatidylinositol (PtdIns) needed to preserve the signaling pathways. A prior study has found that SARS-CoV-2 also affects metabolic pathways like the inositol phosphate metabolism, glycolysis, and oxidative phosphorylation [50]. The dysregulation of those pathways blocks surfactant secretion and alveolar epithelial differentiation. In addition, disrupting the inositol phosphate metabolism may induce neutrophil infiltration and disrupt the lung barrier [50].

In this study, we demonstrated that integrating genomic and clinical features has helped improve the performance of ML models, and implementing the data augmentation approach has addressed the data imbalance issues to enhance the model’s performance further. Similarly, SHAP analysis has helped identify the topmost contributing factors (genes and clinical features) to the model performance that could be biomarkers for predicting disease severity.

## 5. Conclusions

Our study significantly enhances the predictive capabilities for COVID-19 severity by integrating genomic and clinical data. We identified the key contributors to severity prediction by leveraging a sophisticated workflow involving ML techniques, feature selection, data augmentation, and SHAP analysis. We also demonstrated the importance of integrating multi-modality data to improve the performance of prediction models rather than singular modalities. The observed correlations between pre-existing conditions, such as heart disease, lung disease, and hypertension, and the severity of COVID-19 underscore the clinical relevance of our integrative approach. The superior performance of XG in classifying severity groups further validates the efficacy of our predictive models.

The application of SHAP analysis pinpointed specific genes, including COX14, LAMB2, DOLK, SDCBP2, RHBDL1, and IER3-AS1, along with critical clinical features like the absolute neutrophil count and Viremia categories as influential factors in severity prediction. These identified biomarkers offer valuable insights for clinicians for early disease prognosis.

Our study contributes to the evolving understanding of COVID-19 prognosis and provides a foundation for refining clinical decision-making processes. Integrating clinical and genomic data in predictive models holds promise for personalized and timely interventions, ultimately leading to improved patient outcomes. As we continue to navigate the complexities of the pandemic, our findings pave the way for future research and clinical applications aimed at advancing precision medicine in the context of COVID-19 severity prediction.

## Figures and Tables

**Table 1:** Table with a number of samples in the original class and our class definitions.

Original Classification (GSE212041)			Our Classification	
Severity Class Label	Sample Count	Severity Class Description	Class	Sample Count
A1	40	Death	Severe	76
A2	36	Intubated/ventilated, survived		
A3	149	Hospitalized, supplementary O_2_ required, survived	Moderate	149
A4	45	Hospitalized, no supplementary O_2_ required, survived	Mild	74
A5	29	Discharged/Not hospitalized, survived		

**Table 2:** The number of samples in each class, ‘severe’, ‘moderate’, and ‘mild’, before and after data augmentation (using ADASYN).

Class	Number of Samples	
	Pre-Augmentation	Post-Augmentation
Severe	76	120
Moderate	149	149
Mild	74	134

**Table 3:** The evaluation of ML models with 10-fold cross-validation before and after data augmentation for predicting COVID-19 severity. LR: Logistic Regression, XG: XGBoost, NB: Naïve Bayes, SVM: a Support Vector Machine.

Classifier	Before Augmentation		After Augmentation	
	Accuracy (%)	AUC	Accuracy (%)	AUC
LR	43	0.56	81	0.93
XG	40	0.47	95	0.99
NB	31.6	0.45	42	0.70
SVM	50	0.42	55	0.47

**Table 4:** The top canonical pathways from the Ingenuity Pathways Analysis of the top 25% of genes (1324) with the highest SHAP scores.

Top Canonical Pathways	*p*-Value	Overlap
Generic Transcription Pathway	9.68 × 10^−36^	46.5% (199/428)
Immunoregulatory interactions between a Lymphoid and a non-Lymphoid cell	1.75 × 10^−9^	38.1% (77/202)
Mitotic Prometaphase	7.66 × 10^−8^	36.0% (73/203)
Fcgamma receptor (FCGR)-dependent phagocytosis	9.32 × 10^−8^	38.2% (60/157)
Cilium Assembly	2.18 × 10^−7^	35.3% (72/204)

## Data Availability

All analyses were performed using Google Collab (Python version 3.7). All the relevant data generated in the project are made available in the Supplementary Files.

## Supplementary Materials

The following supporting information can be downloaded at https://www.mdpi.com/article/10.3390/diagnostics14121284/s1. Figure S1: Immunoregulatory interactions between a lymphoid and a non-lymphoid cell pathway; Figure S2: Mitotic prometaphase pathway; Figure S3: Fcgamma receptor (FCGR)-dependent phagocytosis pathway; Figure S4: Cilium assembly pathway; Table S1: Gene Expression Data; Table S2: Clinical Data; Table S3: Co-morbidity Data; Table S4: Weighted Gene Features; Table S5: Weighted Clinical Features; Table S6: Weighted Co-morbidity Features; Table S7: Combined Weighted Matrix; Table S8: SHAP Gene Features.
