# Supplementary material for: A Machine Learning Model for the Prediction of COVID-19 Severity Using RNA-Seq, Clinical, and Co-Morbidity Data
Source: Diagnostics (Basel). 2024 Jun 18;14(12):1284. doi: 10.3390/diagnostics14121284 (PMC11202902; doi:10.3390/diagnostics14121284)
Supplement: Supplementary file 1 [file diagnostics-14-01284-s001.zip › Supplementary Figures.pdf]

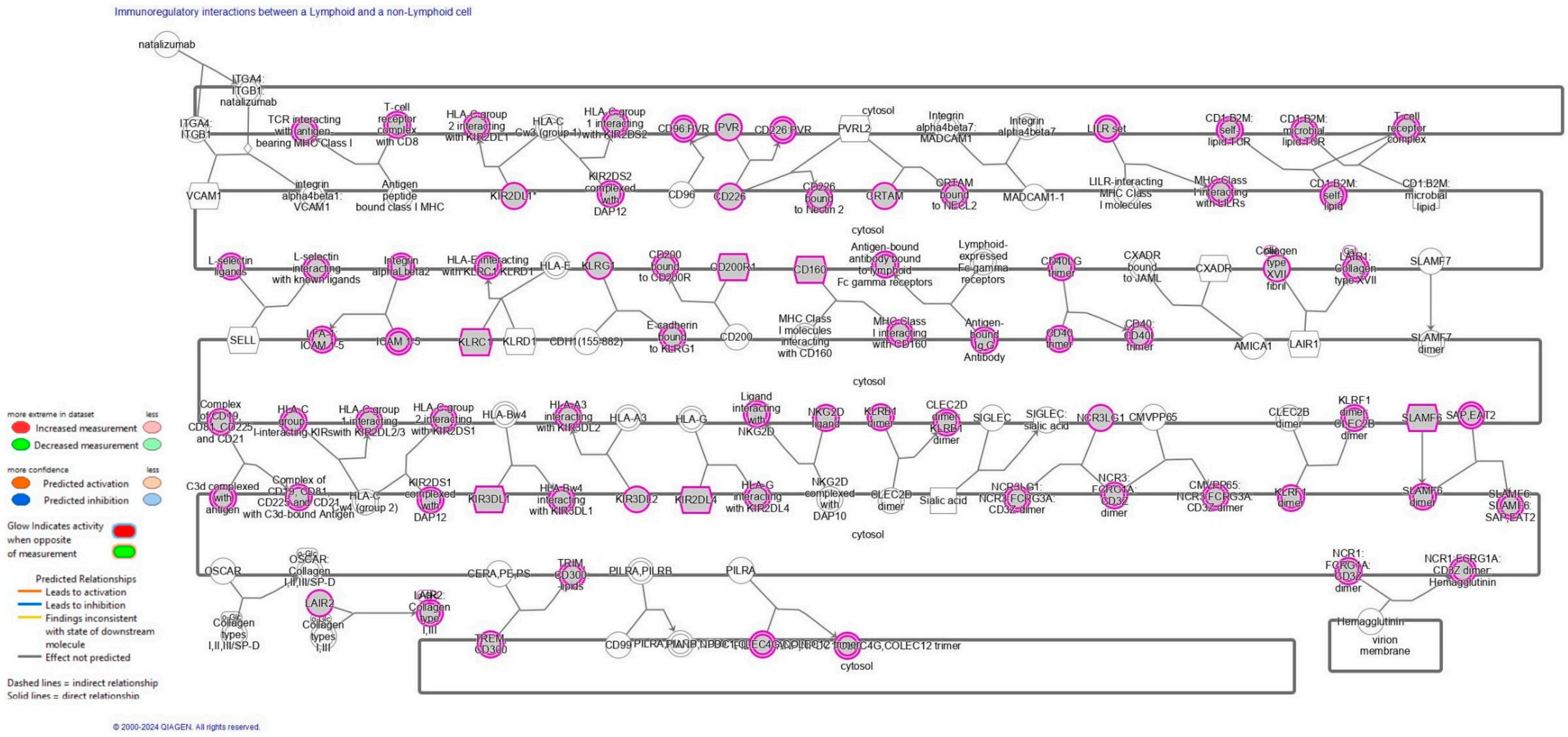

Figure S1: Immunoregulatory interactions between a lymphoid and a non-lymphoid cell pathway

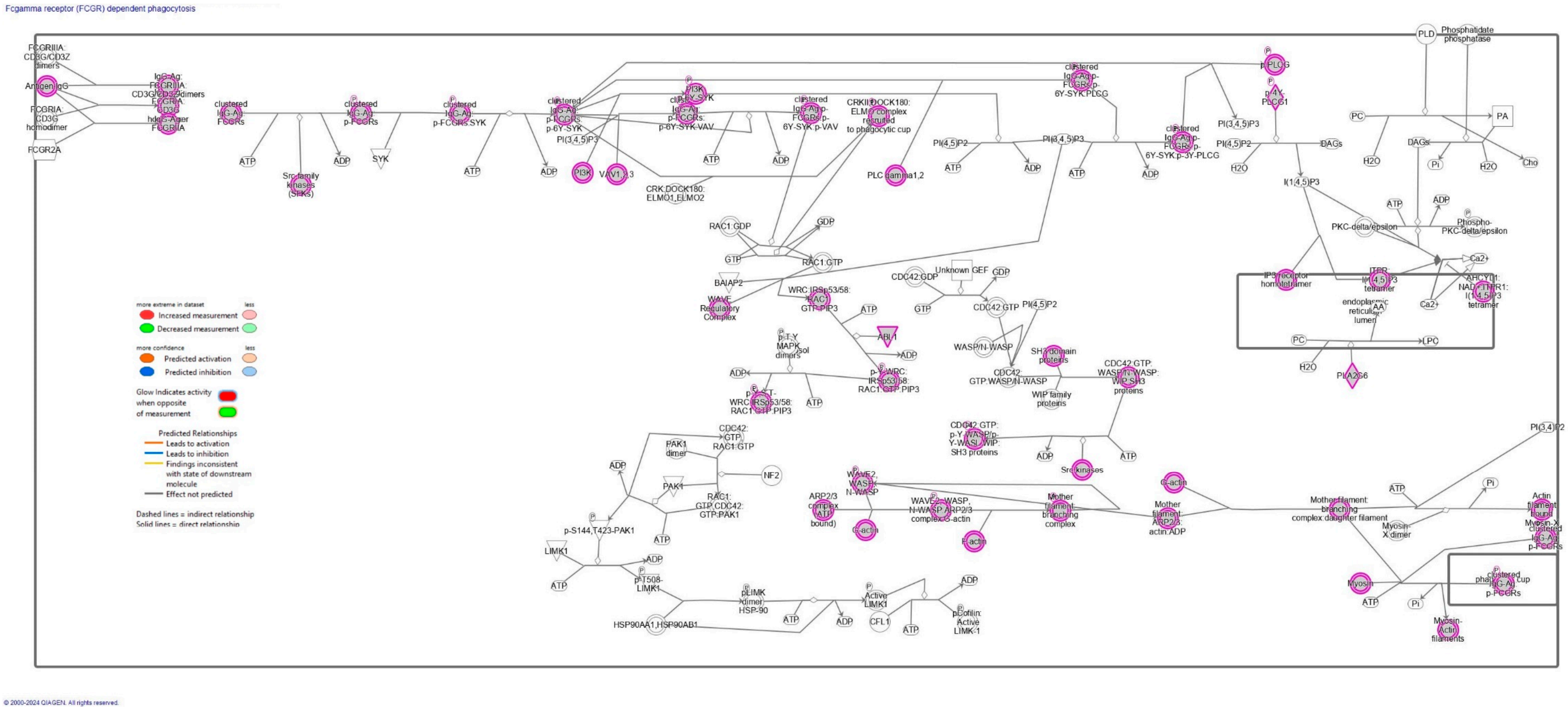

Figure S2: Mitotic prometaphase pathway

Mitotic Prometaphase

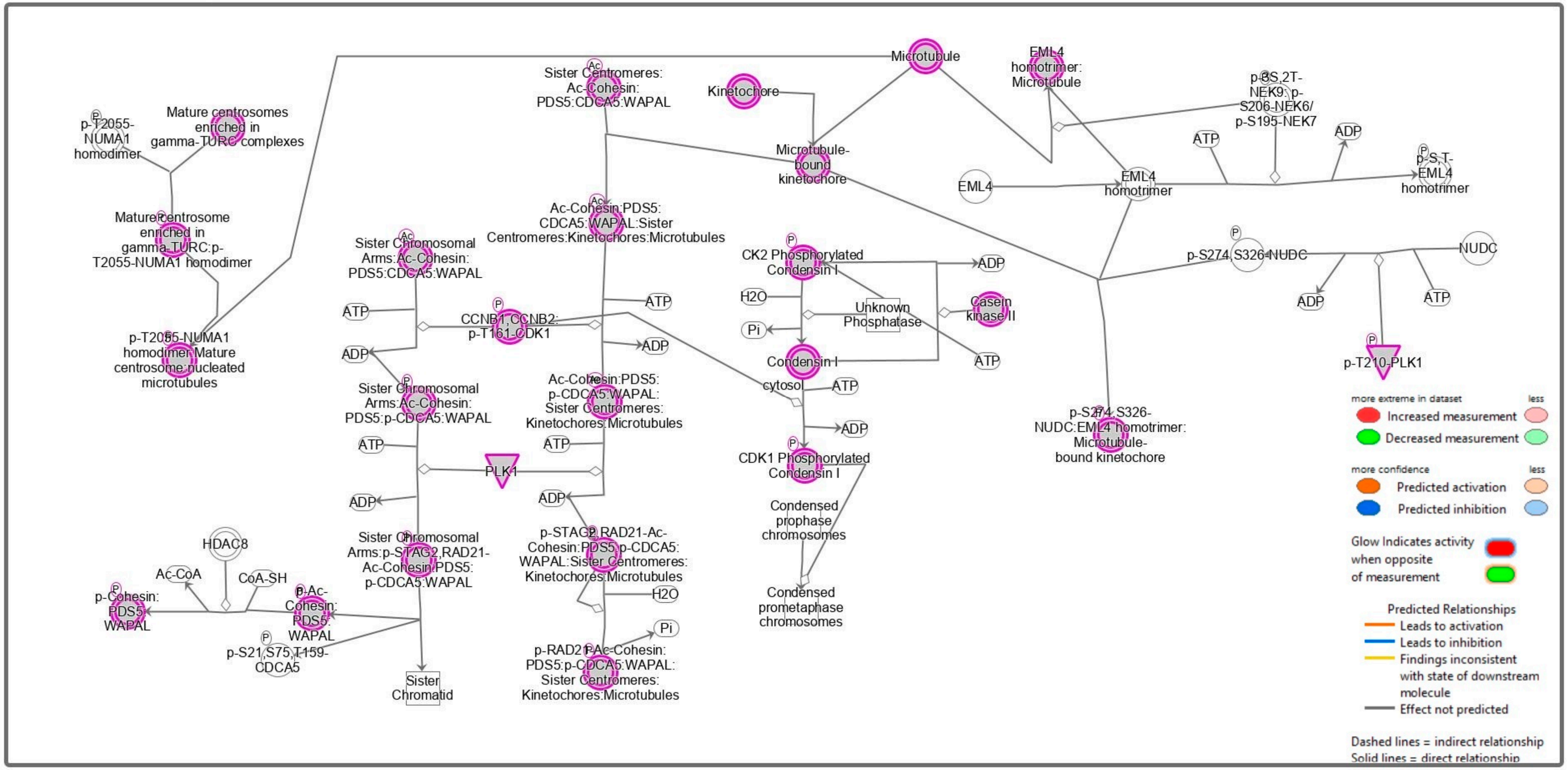

© 2000-2024 QIAGEN. All rights reserved.

Figure S3: Fcgamma receptor (FCGR) dependent phagocytosis pathway

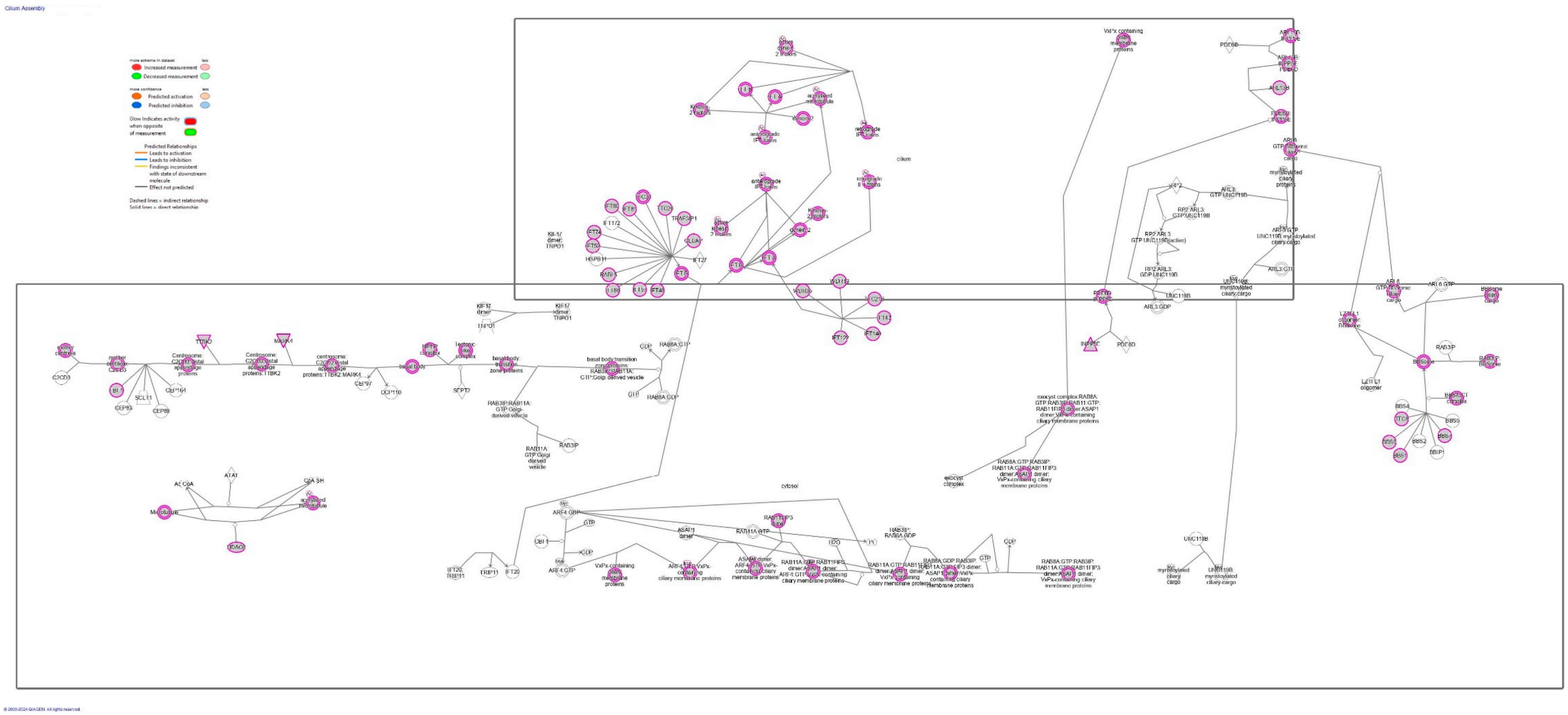

Figure S4: Cilium assembly pathway
