# Supplementary figures and images for: A Machine Learning Model for the Prediction of COVID-19 Severity Using RNA-Seq, Clinical, and Co-Morbidity Data
Source: Diagnostics (Basel). 2024 Jun 18;14(12):1284. doi: 10.3390/diagnostics14121284 (PMC11202902; doi:10.3390/diagnostics14121284)

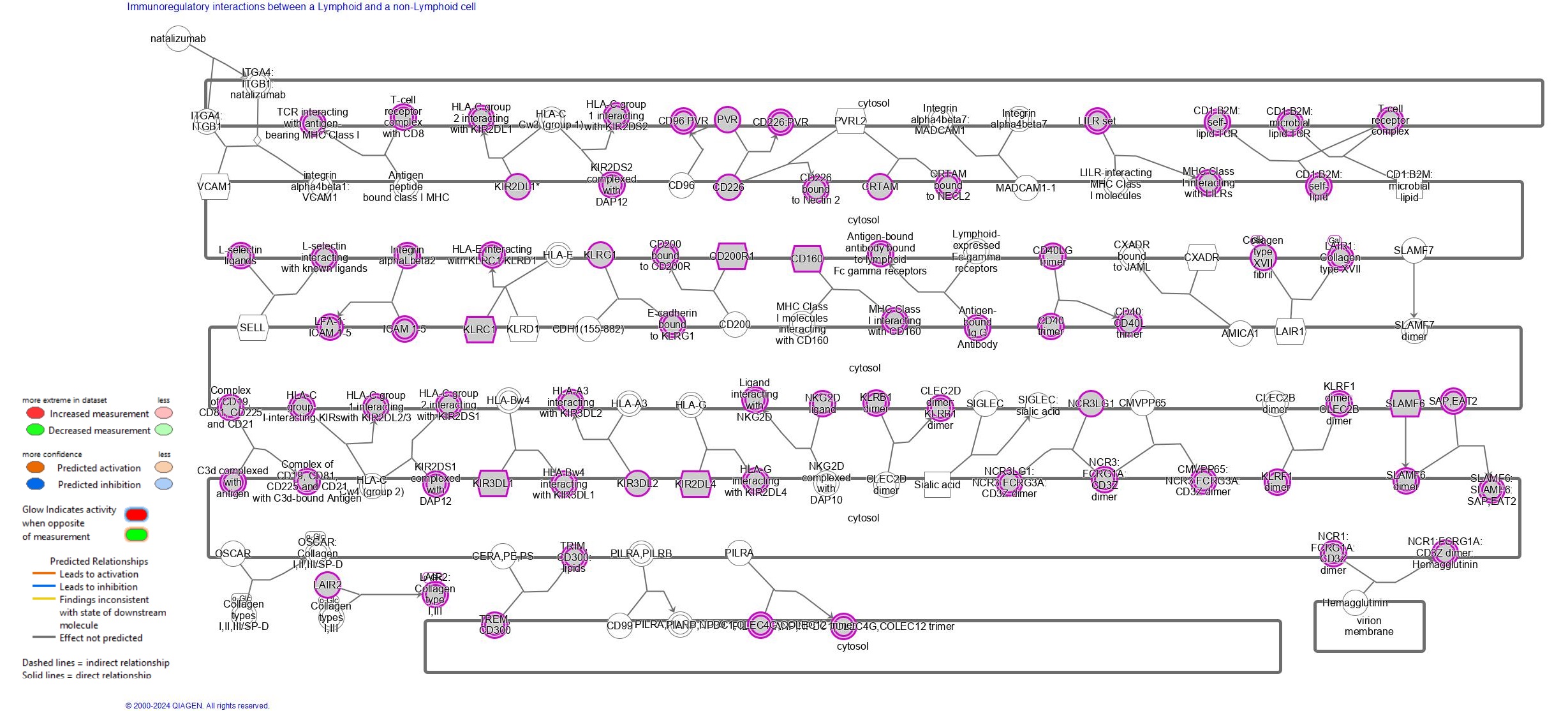

Supplement: Supplementary file 1 [file diagnostics-14-01284-s001.zip › Figure S1.jpg]

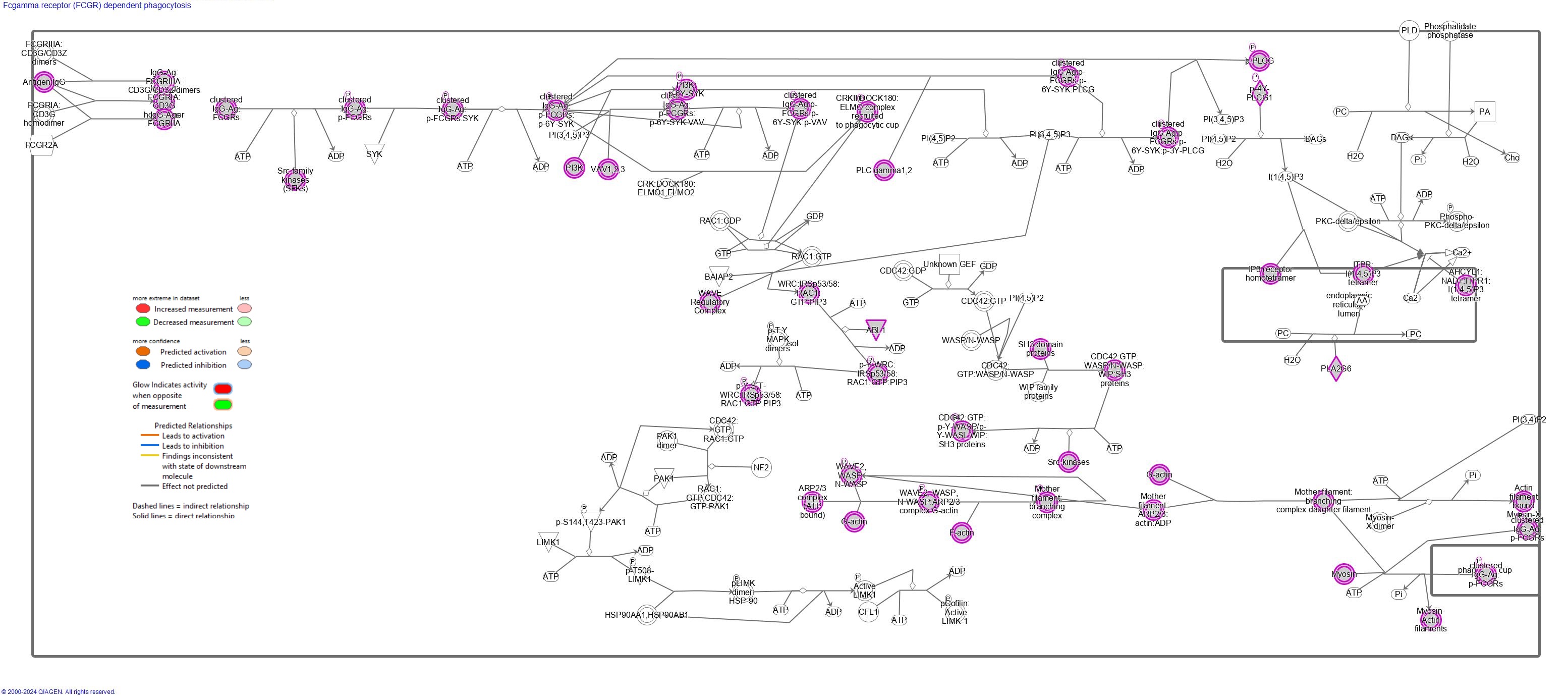

Supplement: Supplementary file 1 [file diagnostics-14-01284-s001.zip › Figure S2.jpg]

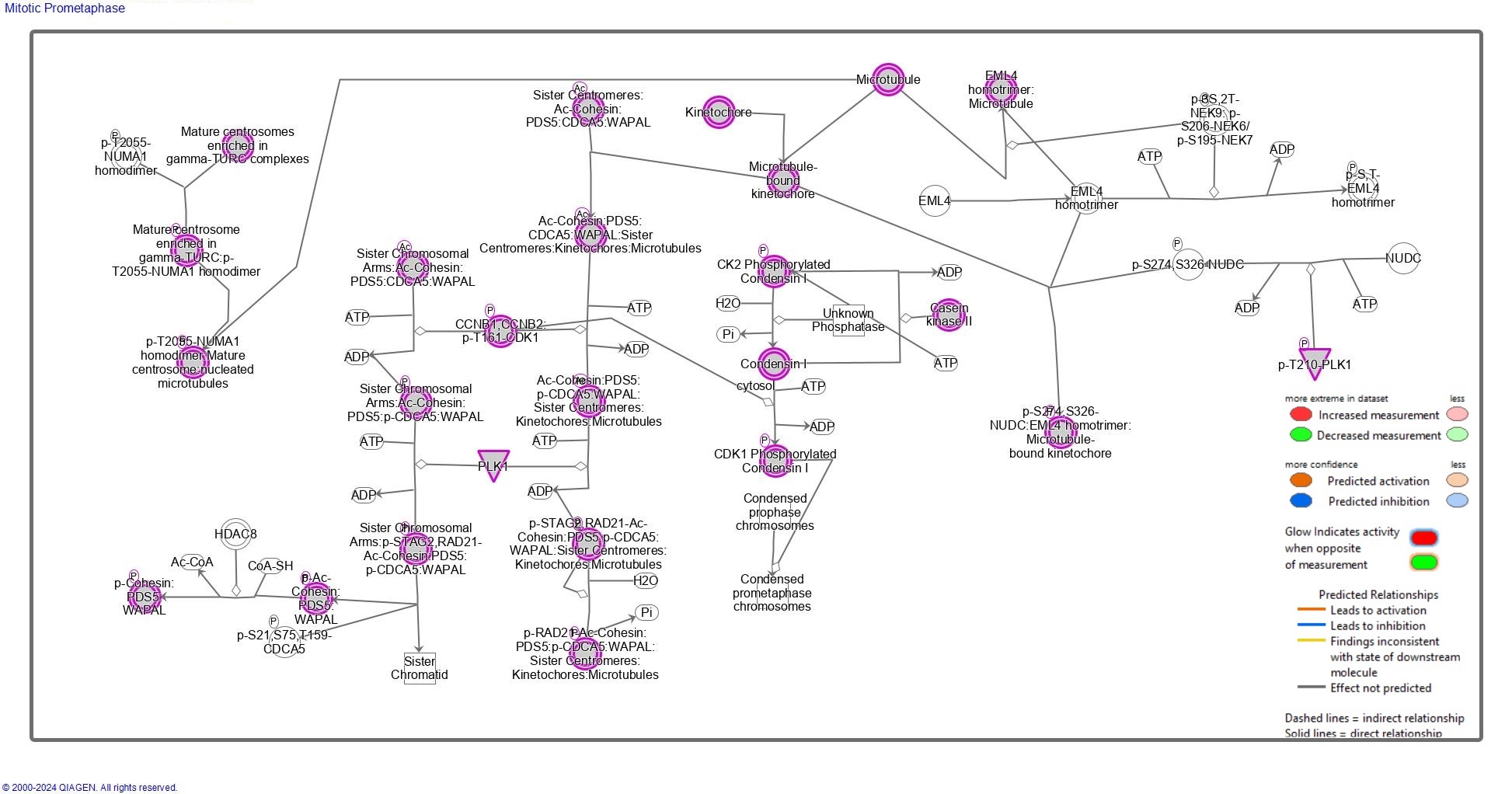

Supplement: Supplementary file 1 [file diagnostics-14-01284-s001.zip › Figure S3.jpg]

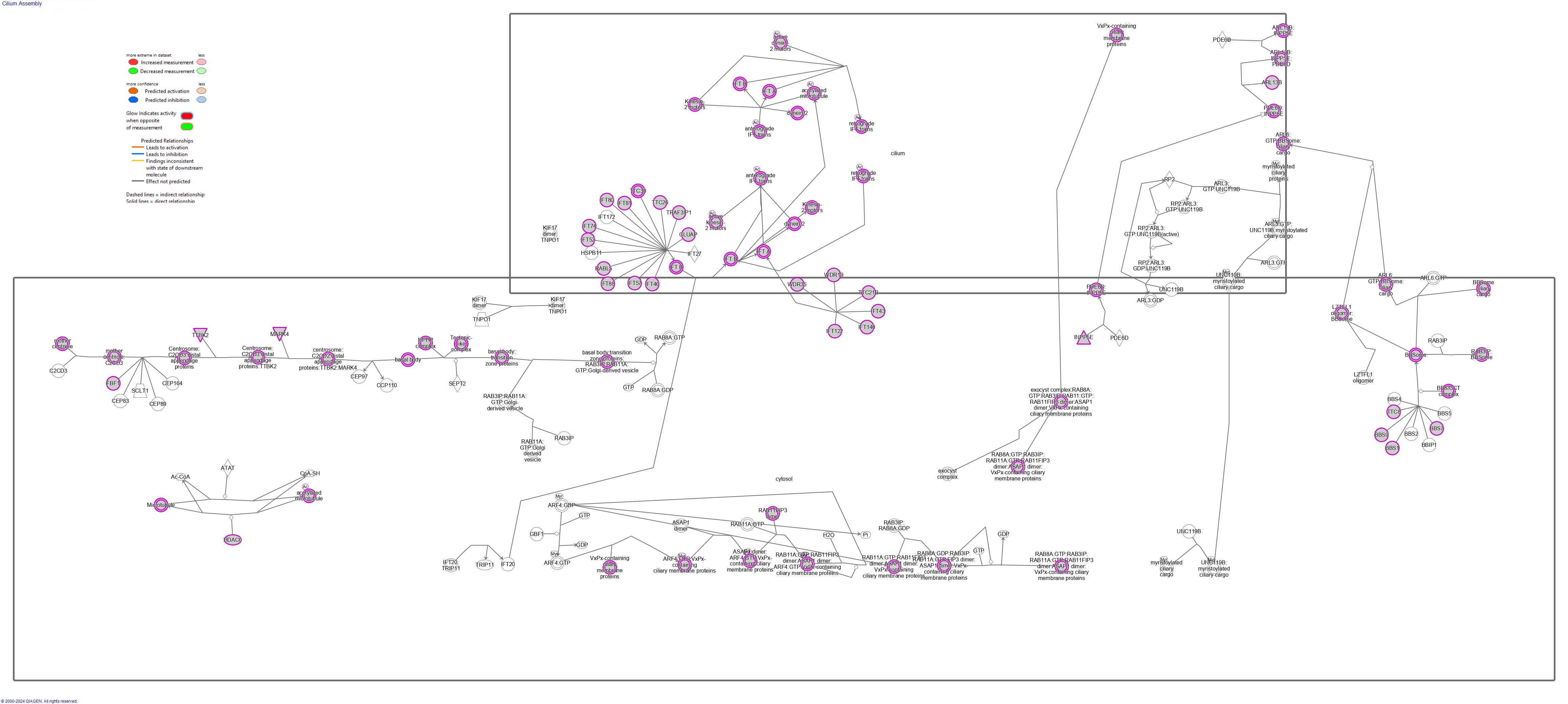

Supplement: Supplementary file 1 [file diagnostics-14-01284-s001.zip › Figure S4.jpg]
